# Nutraceutical Potential of Five Mexican Brown Seaweeds

**DOI:** 10.1155/2019/3795160

**Published:** 2019-12-19

**Authors:** Ana Rocío Múzquiz de la Garza, Mireya Tapia-Salazar, Maribel Maldonado-Muñiz, Julián de la Rosa-Millán, Janet Alejandra Gutiérrez-Uribe, Liliana Santos-Zea, Bertha Alicia Barba-Dávila, Denis Ricque-Marie, Lucía Elizabeth Cruz-Suárez

**Affiliations:** ^1^Universidad Autónoma de Nuevo León, Facultad de Ciencias Biológicas, Programa Maricultura, Ave. Universidad SN, Cd. Universitaria F-67, San Nicolás de los Garza, Nuevo León 66455, Mexico; ^2^Tecnologico de Monterrey, Escuela de Ingeniería y Ciencias, Centro de Biotecnología-FEMSA, Av. Eugenio Garza Sada 2501 Sur, Monterrey, Nuevo León 64849, Mexico

## Abstract

In search of pharmaceutically active products to control type 2 diabetes, five brown seaweeds (*Silvetia compressa*, *Cystoseira osmundacea*, *Ecklonia arborea*, *Pterygophora californica*, and *Egregia menziesii*) from the Northwest Mexican Pacific coast were investigated. Proximate composition and total polyphenol content (TPC) as phloroglucinol equivalents (PGE) were determined for the five seaweed powders and their respective hydroethanolic (1 : 1) extracts. Extracts were screened for their radical scavenging activity (DPPH and ORAC) and glycosidase inhibitory activity. HPLC-DAD, HPLC-MS-TOF, and ATR-FT-IR methodologies were used to identify the most abundant phlorotannins and sulfated polysaccharides in the extracts. Hydroethanolic extracts contained minerals (17 to 59% of the dry matter), proteins (4 to 9%), ethanol-insoluble polysaccharides (5.4 to 53%), nitrogen-free extract (NFE) (24.4 to 70.1%), lipids (5 to 12%), and TPC (2.6 to 47.7 g PGE per 100 g dry extract). *S. compressa* and *E. arborea* dry extracts presented the lowest ash content (26 and 17%, respectively) and had some of the highest phenolic (47.7 and 15.2 g PGE per 100 g extract), NFE (57.3 and 70.1%), and soluble polysaccharide (19.7 and 53%) contents. *S. compressa* and *E. arborea* extracts had the highest antioxidant activity (IC_50_ DPPH 1.7 and 3.7 mg mL^−1^; ORAC 0.817 and 0.801 mmol Trolox equivalent/g extract) and the highest *α*-amylase and *α*-glucosidase inhibitory capacities (IC_50_ 940 and 1152 *μ*g mL^−1^ against *α*-amylase and 194 and 647 *μ*g mL^−1^ against *α*-glucosidase). The most abundant phlorotannins identified in the extracts were phloretol, fucophloroethol, and two- and three-phloroglucinol unit (PGU) phlorotannins. Laminarin, fucoidan, and alginate were among the sulfated polysaccharides identified in the extracts. The bioactivities of *S. compressa* and *E. arborea* extracts were mainly related with their contents of three PGU phlorotannins and sulfated polysaccharides (e.g., fucoidan, laminarin, and alginate). These results suggest *S. compressa* and *E. arborea* are potential candidates for food products and nutraceutical and pharmaceutical preparations, and as additives for diabetes management.

## 1. Introduction

Globally, there are approximately 350 million people currently suffering from diabetes (http://www.who.int/mediacentre/factsheets/fs312/en/). This number could potentially double by 2030, which would make diabetes the seventh most prevalent cause of death worldwide [1]. Ninety percent of diabetes cases are type 2 diabetes mellitus, a complex disorder characterized by hyperglycemia and associated with high oxidative stress caused by the production of reactive oxygen species (ROS) [2]. Dietary starch degradation by glycosidases is the major source of glucose in the blood [3, 4]. Dietary polysaccharides are hydrolyzed by *α*-amylase to oligosaccharides and disaccharides, which are further hydrolyzed to monosaccharides by *α*-glucosidase (a membrane-bound intestinal enzyme that aids glucose liberation by acting as a catalyst in the hydrolysis of the *α*-glycosidic bond found in oligosaccharides). Liberated glucose is absorbed from the intestine and contributes to postprandial hyperglycemia. Glycosidase inhibitors prevent or delay the hydrolysis or absorption of carbohydrates and reduce postprandial hyperglycemia, making such inhibitors useful in the management of type 2 diabetes [3, 5]. Thus, antidiabetic therapies that limit the postprandial increase of blood glucose levels after a mixed carbohydrate diet, such as the use of glycosidase inhibitors, are particularly relevant in diabetes prevention and control [6, 7]. Certain synthetic antidiabetic drugs that act by inhibiting *α*-amylase and *α*-glucosidase activity (e.g., acarbose, miglitol, and voglibose) are currently available, but they are associated with undesirable side effects, such as liver toxicity and adverse gastrointestinal symptoms [6, 8, 9]. Such adverse effects might be caused by the excessive inhibition of pancreatic *α*-amylase, resulting in abnormal bacterial fermentation of undigested carbohydrates in the colon [9]. Lower inhibitory effect against *α*-amylase activity and stronger inhibition activity against *α*-glucosidase can be an ideal approach for managing hyperglycemia with minimal side effects [9]. Thus, there is a substantial need for natural *α*-amylase and *α*-glucosidase inhibitors that have no adverse or unwanted secondary effects [6, 7]. A number of studies suggest that marine compounds may be convenient alternatives [8].

Seaweeds are rich in dietary fibre, polyphenolic compounds, unsaturated fatty acids, and minerals, among other compounds, many of which can be beneficial to human health, including in managing diabetes [10–13]. In fact, dietary consumption of seaweed*s* was associated with a low incidence of diabetes in Korean men [14]. Particularly, numerous brown seaweed crude extracts (acetonic, aqueous, methanolic, and ethanolic) have shown to possess antioxidant [15, 16] and antidiabetic (e.g., *α*-amylase and *α*-glucosidase inhibition) activities [14, 17–19]. These activities are related to the presence of phenolic compounds, phlorotannins, pigments, tocopherols, polysaccharides, fatty acids, and peptides in the seaweed extracts [15, 20–28]. The main compounds reported as potent *α*-amylase and *α*-glucosidase inhibitors are as follows: phloroglucinol derivatives such as dieckol, 8,8′-bieckol, phlorofucofuroeckol, fucophloroethol, and phlorotannins with low PGU [23, 25, 29–31], fucoidan [3, 32, 33], and oleic, linoleic, and eicosapentaenoic acids [24].

On the other hand, the major compounds contributing to overall antioxidant activity in seaweed are phenolic compounds and polysaccharides, the latter alone or associated with other components such as polyphenols, amino acid, protein, lipids, and nucleic acids residues, and sometimes polysaccharide conjugates [28, 34]. Hence, TPC and polysaccharides, in combination with *in vitro* antioxidant assays, are typically used to screen for seaweed antioxidant activity [34–36]. Previous studies have found a positive correlation between high TPC content and high radical-scavenging capacity for seaweed extracts [34, 35, 37]. Powerful antioxidant bioactivity was observed in dieckol (A), 6-6′-bieckol (B), and fucodiphlorethol G (C) separated and refined from *Ecklonia cava*, an edible marine alga collected at Jeju Island [38]. Sulfated polysaccharides also possess excellent *in vitro* antioxidant activity, including both radical-scavenging capacity and metal chelating ability [34, 39–41]. Therefore, substantial evidence exists to support the claim that seaweed extracts and their fractions could act as functional ingredients in foods used to control hyperglycemia [19, 27].

The coastline of Baja California, Mexico, is an abundant source of seaweeds with a broad diversity of species, some of which have been under commercial exploitation since the 1960s (e.g., *Macrocystis pyrifera*, *Gelidium robustum*, *Chondracanthus canaliculatus*, and *Gracilariopsis lemaneiformis*) to obtain alginate, agar, and carrageenan [42, 43]. In contrast, other commercially available edible brown seaweed species, such as *Silvetia compressa* (J. Agardh) (De Toni 1985), *Cystoseira osmundacea* (Turner) (C. Agardh 1820), *Ecklonia arborea* (Areschoug 1876), *Pterygophora californica* (Ruprecht, 1852), and *Egregia menziesii* (Turner) (Areschoug 1876), are incipiently being used as supplements for human and animal consumption, as well as in the elaboration of cosmetic products (https://www.bajakelp.net).

Although proximal composition, polyphenol content, and DPPH antioxidant activity have been previously reported for *Ecklonia arborea* (formerly *Eisenia arborea*) [15, 44–46], this information is incomplete or unavailable for *S. compressa* [46–48], *E. menziesii* [47], *C. osmundacea*, and *P. californica* from the Baja California coast. Neither the antidiabetic potential of these five seaweeds (*α*-amylase and *α*-glucosidase inhibition activities) nor the chemical composition of their hydroethanolic extracts has been previously reported. This study seeks to screen the antioxidant and glycosidases inhibiting potential of hydroethanolic extracts from these five brown seaweeds, as well as to identify the probable active compounds: phlorotannins (using HPLC-DAD and HPLC-MS-TOF methodologies) and sulfated polysaccharides (using ATR/FT-IR methodologies). Through this process, this study hopes to identify a potential commercial value for these five macroalgae native to Mexico's Baja California coast and to find candidates for the development of pharmaceutically active products that can control type 2 diabetes.

## 2. Materials and Methods

### 2.1. Chemicals and Reagents

Potato soluble starch (S-2360), *α*-amylase from porcine pancreas (A-3176, 5 MU), acarbose (A-8980), rat intestinal acetone powders (I-1360), 4-nitrophenyl-*α*-D-glucopyranoside (N-1377), Folin–Ciocalteu reagent (F-9252), sodium carbonate (Na_2_CO_3_) (S-2127), phloroglucinol (P-3502), gallic acid (G-7384), 2,2-diphenyl-1-picrylhydrazyl (DPPH) (D-9132), 3,5-dinitro-2-hydroxybenzoic acid (DNS) (D-0550), HEPES (4-(2-hydroxyethyl)-1-piperazine ethanesulphonic acid) buffer solution (H3375), fucoidan from *F. vesiculosus* (F8190), laminarin from *L. digitate* (L9634), sodium alginate from brown algae (w201502), fluorescein (F6377), and 2,2′-azobis (2-methylpropionamidine) dihydrochloride (AAPH, 4409914) were purchased from Sigma Aldrich Co. (St. Louis, MO, USA). Ethanol (absolute) was obtained from Desarrollo de Especialidades Químicas SA de CV (Monterrey, NL, Mexico). Distilled water was purchased from Garvy SA de CV (Monterrey, NL, Mexico); HPLC-grade water (4218-03) was obtained from J. T. Baker Co. (Center Valley, PA, USA). Formic acid 88% (A11-8P) and HPLC-grade methanol (A452-4) were purchased from Fisher Scientific Co. (Pittsburgh, PA, USA). EDTA (502-092) was purchased from LECO Co. (St. Joseph, MI, USA).

### 2.2. Seaweeds

Five edible dehydrated seaweed samples were obtained from BajaKelp (https://www.bajakelp.net) in Ensenada, Baja California, Mexico. Samples of *S. compressa* (formerly *Pelvetia compressa*), *C. osmundacea*, *E*. *arborea*, *P*. *californica*, and *E. menziesii* were collected from December 2014 to January 2015 at La Escalera, Baja California Peninsula, Mexico (31°30′ 59.1″ N-116°38′ 51.1″ W). Seaweed fronds were washed with seawater to remove sand and epiphytes, drained on clotheslines, and sun-dried inside a greenhouse in Ensenada, Baja California. Samples were ground at our lab in Monterrey, Nuevo León (Pulvex 200, CDMX, MEX), with a 500 *μ*m sieve and vacuum-packed until use. Identity of all five seaweed species was confirmed using taxonomic keys [49].

### 2.3. Proximal Composition of Seaweeds

Moisture and ash content were determined using 930.15 and 942.05 AOAC [50] procedures, respectively. Crude protein (Nx6.25) was quantified by AOAC 930.03 method using a nitrogen analyzer (Truspec CHN, Leco Corporation, St. Joseph, MI, USA). Crude lipid was determined using the Bligh and Dyer method, following the methodology described by Li et al. [51]. Nitrogen-free extract (NFE) or carbohydrate value was estimated from the difference between dry weight (100) and the sum of total lipids, protein, and ash (minerals). Data were expressed as % of seaweed dry matter (DM).

### 2.4. Preparation of Hydroethanolic Extracts

For phenolic compound extraction, we used the methodology described by Xi et al. [52]. For this, 10 g of seaweed powder and 200 mL aqueous ethanol 50% were mixed in a 250 mL Erlenmeyer flask and sonicated (Ultrasonic cleaner 50HT, VWR International, West Chester, PA, USA) for 30 min. Samples were then incubated at 70°C with constant agitation at 100 rpm (Shak-R-bath, Lab-line, Melrose Park, IL, USA) for 2 h. Subsequently, samples were cooled down to room temperature and centrifuged at 2500 rpm (IEC Centra MP4R, International Equipment Company, Needham, MA, USA) for 15 min. The supernatant was transferred to an evaporator (Rocket Synergy evaporator, Genevac, Ipswich, UK) to remove ethanol and water. Then, crude extracts were stored at −80°C until analysis. To obtain the hydroethanolic extraction yield, a 1 mL extract sample was placed in preweighed test tubes, weighed, evaporated in a hot air oven (Shel Lab 1330 FX, Sheldon Manufacturing Inc., Cornelius, OR, USA) at 130°C for 60 min, cooled down in a desiccator, and weighed again. The solid extraction yield was calculated as

(1) $$\begin{array}{c} Y\left(\%\right)=\frac{g\text{ of dry extract obtained from}\;1\text{ mL sample }*\;\left(\text{mL of total extract}\right)}{g\text{ dry seaweed}}\;*\;100. \end{array}$$

Three extractions were run for each seaweed sample.

### 2.5. Total Phenolic Content (TPC)

TPC was measured using the Folin–Ciocalteu method [53], where 200 *μ*L of each liquid seaweed extract (eventually after some dilution) was transferred into a 1.5 mL Eppendorf tube and mixed with 50 *μ*L of Folin–Ciocalteu (2 M) reagent and 750 *μ*L of sodium carbonate (7.5% w/v). The mixture was homogenized for 15 s in a vortex (Standard Heavy-Duty Vortex Mixer VWR, Radnor, PA, USA), after which the tubes were allowed to stand in complete darkness for 2 h and centrifuged at 2500 rpm for 15 min. 200 *μ*L of each sample reaction was transferred to a 96-well microplate, and absorbance was registered at 620 nm in a microplate reader (Epoch 2, BioTek Instruments Inc, Winooski, VT, USA). TPC was determined by comparison of the values obtained with the calibration curve of phloroglucinol using a seven-point calibration curve (0 to 1.25 mg mL^−1^). Results were expressed as g of phloroglucinol equivalents (PGE) per 100 mL of dry extract or per 100 g of dry seaweed by considering an average of 140 mL hydroethanolic extract obtained from a 10 g seaweed meal sample.

### 2.6. Proximal Composition of Seaweed Extracts

These analyses were performed for all extracts following the methods described above. In addition, polysaccharide content of each extract was determined using the method described by Tako et al. [54]. Ethanol (4 : 1 v/v ratio) was added to the hydroethanolic liquid extract to precipitate the polysaccharides. After that, samples were centrifuged for 10 min at 3500 rpm, the supernatant filtered, and the precipitate dried overnight at 60°C and weighed. Data were expressed as % of dry extract.

### 2.7. Antioxidant Capacity

DPPH free radical-scavenging activity was determined following the methodology described by García-Becerra et al. [55]: 100 *μ*L of the extract serial dilutions using ethanol 50% (1 : 1) and 100 *μ*L of methanol-DPPH solution (20 *μ*g mL^−1^) were transferred in a 96-well microplate. Samples were incubated for 30 min at room temperature, and absorbance was measured at 550 nm (Epoch 2, BioTek Instruments Inc., Winooski, VT, USA). The IC_50_ (i.e., the concentration of antioxidant required to cause a 50% reduction in the original DPPH concentration) was calculated using a dose-inhibition curve in a linear range by plotting the extract concentration (mg mL^−1^) versus the corresponding scavenging effect. The oxygen radical absorbance capacity (ORAC) was determined as described by Ou et al. [56]. Briefly, aliquots of 25 *μ*L seaweed extract were diluted in 75 mM phosphate buffer and transferred to a 96-well round opaque bottom microplate. Reaction fluorescence was measured using a Synergy HT microplate reader with an auto dispenser (BioTek, Instruments, Winooski, VT, USA) at a wavelength of 485 nm (excitation) and 580 nm (emission) at intervals of 70 s for 70 minutes. Equipment was programmed to dispense 200 *μ*L of 0.96 *μ*M fluorescein and 75 *μ*L of 95.8 *μ*M AAPH (2,2ʹ-azobis (2-amidinopropane) dihydrochloride) used as free radical. Protective effects of experimental and control samples were calculated by subtracting the net integrated area under the curve (AUC) of the control from that of the experimental sample (AUC sample–AUC control). Results were quantified using Trolox as standard and expressed as Trolox equivalents (TE) millimolar concentration: mmol TE/g dry mass seaweed extract.

### 2.8. Glycosidase Inhibitory Enzyme Activities

Inhibitory activity for *α*-amylase was obtained following the method of Kazeem et al. [57] by using different concentrations of the dry extract (200, 400, 600, 800, 1000, and 1200 *μ*g mL^−1^) and using acarbose (200, 400, 600, 800, 1000, and 1200 *μ*g mL^−1^) as a positive control. Briefly, tubes containing 250 *μ*L acarbose (1 mg mL^−1^) or seaweed extract along with a sodium phosphate buffer (0.02 M pH 6.9) or 500 *μ*L *α*-amylase solution in a phosphate buffer (with 0.006 M de NaCl, 13 U mL^−1^) were preincubated at room temperature for 10 min with 250 *μ*L 1% starch solution (sodium phosphate buffer). Tubes were then boiled for 5 minutes after adding 1 mL of DNS (method of Miller, 1959) [58] to stop the reaction. Samples were diluted in 10 mL of distilled water, and absorbance was measured at 540 nm (UNICO S1200, Dayton, NJ, USA). *α*-Amylase inhibition was calculated using equation (2), where *K* = absorbance of control blank, *S*1 = absorbance of sample with enzyme, and *S*0 = absorbance of sample without enzyme. Inhibitory activity was expressed as the concentration allowing half maximal inhibitory activity (IC_50_). The IC_50_ value of each extract was determined from the plots of percent inhibition versus inhibitor concentration.

(2) $$\begin{array}{c} \text{inhibition}\left(\%\right)=\frac{K-\left(S1-S0\right)}{K}*100. \end{array}$$

The *α*-glucosidase test was performed using different extract concentrations (200, 400, 600, 800, 1000, and 1200 *μ*g mL^−1^), as described by Mayur et al. [59]. Rat intestinal acetone powder was mixed with distilled water (10 mg mL^−1^) and centrifuged for 10 min at 10,000 rpm. The assay buffer was 100 mM HEPES at pH 6.8, and the substrate was 2 mM 4-nitrophenyl-*α*-d-glucopyranoside. The assay constituents were added to 96-well microplates in the following order: 100 *μ*L of enzymatic solution (10 mg rat intestinal acetone powder per 1 mL distilled water), then 50 *μ*L of acarbose or seaweed extract or buffer, and finally 50 *μ*L of substrate. Samples were incubated at 25°C for 2 hours. Absorbance was recorded at the beginning and end of the incubation time at 405 nm. *α*-Glucosidase inhibition was calculated using equation (2). Acarbose (1 mg mL^−1^) was used as a positive control. The inhibitory activity was expressed as the concentration required to obtain half maximal inhibitory activity (IC_50_). Extracts' IC_50_ values were determined from the plots of percent inhibition versus inhibitor concentration.

### 2.9. Preliminary Identification of Possible Active Compounds

#### 2.9.1. Identification of Major Phlorotannins in Seaweed Extracts

Identification of major phlorotannins was performed by high-performance liquid chromatography (Agilent Technologies 1200 series chromatograph, Santa Clara, CA, USA) coupled with a diode array detector (HPLC-DAD). The analysis was carried out on a Luna C18 column (250 mm × 4.6 mm, 5 *μ*m particle size, Phenomenex, Macclesfield, UK) at a flow rate of 0.8 mL min^−1^ (injection volume 10–20 *μ*L). The isocratic mobile phase consisted of water acidified with 1% of formic acid. Spectral data from all peaks were accumulated in the range 230–550 nm, and chromatograms were recorded at 270 nm. Chromatographic data were collected using Chemstation for LC software (Hewlett-Packard-Agilent Technologies, Waldbronn, Germany). Phloroglucinol was used as a standard to quantify bioactive compounds by linear regression. The concentration of bioactive phenolic compounds (*n* = 3) was expressed as *μ*g of PGE per mL extract, using equation (3) as the calibration curve (*R*^2^ = 0.9996):

(3) $$\begin{array}{c} y=24112x-2.0812. \end{array}$$

The same chromatographic conditions described above were used to identify bioactive compounds by HPLC coupled to time-of-flight mass spectrometry (MS-TOF) technique (G1969A, Agilent Technologies 1100, Santa Clara, CA, USA). Mass spectra were collected using electrospray source in the positive mode (ESI+) under the following conditions: *m*/*z* range 100 to 1500, nitrogen gas, gas temperature 300°C, drying gas flow rate 8 L/min, nebulizer pressure 20 psi, capillary voltage 4000 V, and fragmentor voltage 70 V. Extracted ion chromatograms were obtained using the Analyst QS 1.1 software (Applied Biosystems, Carlsbad, CA, USA) and considering the accurate mass from each compound or its adducts with Na or K with an error range of 0.01 units. Mass spectra were used to identify the different phenolic compounds based on their fragmentation patterns, which were subsequently compared to previous studies [21, 22] and verified.

#### 2.9.2. Sulfated Polysaccharides

Identification of the principal functional groups in sulfated polysaccharides present in the dry hydroethanolic seaweed extracts was done using a Fourier transform infrared (FT-IR) spectroscopy (Perkin Elmer Spectrum-ONE, Shelton, CT, USA, equipped with an attenuated total reflectance sampling device (ATR)). Spectra samples (including the standards alginic acid, laminarin, and fucoidan) with any previous preparation were recorded between the 4000 and 650 cm^−1^ range (10 scans, pressure 90–100 Gauges at room temperature) and the analytical spectral range from 1700 to 650 cm^−1^ in transmittance mode. A background spectrum air was scanned under the same instrumental condition before each series of measurements.

### 2.10. Statistical Analysis

Each variable was determined in triplicate. All reported data were expressed as mean ± standard deviation and submitted to one-way ANOVA statistical analysis and Tukey's posttest (*P* < 0.05) using SPSS Software, version 20 (IBM Corporation, Armonk, NY, USA). Correlations (r-Pearson correlation coefficient) between activities and chemical compounds present in seaweed extracts were determined using Microsoft Excel.

## 3. Results and Discussion

### 3.1. Proximal Composition and Total Polyphenol Content in Seaweeds

The chemical composition of the seaweed samples presented in Table 1 agreed with previous information reported for brown seaweeds [60, 61]. *S*. *compressa*, *E*. *arborea*, and *E. menziesii* presented the highest protein contents, ranging from 10 to 12% DM, agreeing with the values (8–12%) reported for the same species in previous studies [45, 47]. In contrast, *C. osmundacea* and *P. californica* presented a lower protein content (9-10% DM), but no previous studies were available for comparison. Lipid content in all seaweed species was low, as expected: *S. compressa* had the highest lipid content (2.93% DM), followed by *C. osmundacea* (1% DM), while *E. arborea*, *P. californica*, and *E. menziesii* were very poor in lipids (0.6% DM). These values coincide with those previously reported for *E. arborea* (0.19%) [45] and *E. menziessi* (0.14%) [47], but not for *S. compressa*, which had twice the content (1.46%) reported by Guerra-Rivas [47]. Ashes and carbohydrates were the major compounds in all seaweeds, ranging between 23–34% and 54–65% DM, respectively. *E. arborea* and *S. compressa* presented the lowest ash (23 and 25%) and the highest NFE (62 and 65%) contents, respectively, while the other three algae were richer in ashes (30–34%) and poorer in NFE (54–59%). These results coincide with previously reported values for *E. arborea* (27.2 ash and 55.30% NFE +5.04% fibre) [45], *S. compressa* (15.9% ash and 50.6% NFE +6.47% fibre), and *E. menziessi* (28.9% ash and 40 NFE +7.2% fibre) [47]. They are also in accordance with the high total dietary fibre content recently reported by Tapia-Salazar et al. [46] for *S. compressa* and *E. arborea* (59 and 55% DM, respectively).

In the case of seaweed TPC, *S. compressa* and *E. arborea* also had the highest concentrations (8.32 and 5 PGE per 100 g dry seaweed), followed by *C. osmundacea* (4%), *P. californica* (1.9%), and finally *E. menziesii*, which distinguished itself for having the lowest content (0.53%) (Table 1). *E. arborea* TPC was in the high range compared to Japanese *E. arborea* and *E. bicyclis*, 60% methanol extracted (2.7–6.6 g PGE per 100 g dry seaweed) [62]. TPC in *S. compressa* was higher than the previously reported concentration for the same species (formerly known as *Pelvetia fastigata*) extracted with 80% methanol (5.2 to 6 g PGE per 100 g dry seaweed) [63]. *C. osmundacea* presented higher TPC when compared to methanol extracts of *C. neglecta* and *C. osmundacea* (1.37 and 1.60 g PGE per 100 g dry seaweed) [63]. TPC of *Egregia menziesii* was within the values previously reported for methanol extracts (0.36 to 2.16 g PGE per 100 g dry seaweed) [63]. In the case of *P*. *californica*, no previous studies for TPC were found. Differences in TPC between this and previous studies could be attributed to several factors such as seaweed collection area, season, drying method, solvents, and extraction conditions. In summary, among the five species studied, *S. compressa* and *E. arborea* had the highest carbohydrate and phenolic contents.

### 3.2. Seaweed Hydroethanolic Extracts

Extraction yield, proximal composition, and total phenolic content of hydroethanolic extracts were significantly different between seaweed species (*P* < 0.001, Table 2). The best extraction yield (32.8% of dry seaweed) was obtained from *E. arborea*, while the mass yield for *E*. *menziesii*, *P*. *californica*, *C*. *osmundacea*, and *S. compressa* was very similar and ranged from 17.5 to 20.3%. Under the extraction conditions used in this study, 50% ethanol efficiently extracted inorganic and organic polar compounds, resulting in seaweed extracts consisting of minerals (17 to 59% DM), proteins (4.23 to 9.30% DM), lipids (5 to 12% DM), soluble polysaccharides (5.4 to 53.2% DM), NFE (16.9 to 70.1% DM), and polyphenols (2.6 to 47.7% PGE DM). These results fall within the ranges previously reported for 60% ethanol extracts from *L*. *cichorioides*, *C*. *costata*, and *F. evanescens* [64] (24 to 60% ash, 4 to 8% protein, 1.4–10.1% PGE, 23 to 67% NFE, 3.6–12% lipophilic matter), except for polyphenol compounds, which were higher in our extracts. Nevertheless, direct comparison to other studies is complicated due to potential differences in solvent type, concentration, seaweed/solvent volume ratio, extraction methods, and species tested, which influence the extracts moisture, minerals, polysaccharide and polyphenol content, and the extraction yield.

*E. arborea* and *S. compressa* extracts presented a proximal composition very similar to that of the dried seaweed. These seaweed extracts presented the highest polysaccharide (53 and 20%, respectively), NFE (70.1 and 57.3%, respectively), phenolic (15.2 and 47.7% PGE, respectively), and protein (11.1 and 10.4%) contents. In the case of *C. osmundacea* and *E. menziesii* extracts, minerals were the predominant compounds and the lipophilic matter content was elevated. Finally, *P. californica* extract had a mild carbohydrate, low phenol content, and high lipid content (Table 2).

### 3.3. Antioxidant Activity

The five macroalgae extracts were evaluated as radical scavengers against DPPH, showing significant differences (*P* < 0.0001) among IC_50_ values (Table 3): *E. menziesii* extract was the most effective (IC_50_ 0.93 mg mL^−1^), followed by *E. arborea* and *S. compressa* extracts showing intermediate IC_50_ values (1.7 and 3.7 mg mL^−1^), and the least potent were *C. osmundacea* and *P. californica* extracts with the lowest scavenging activities (IC_50_ 4.2 and 8.1 mg mL^−1^). None of the extracts evaluated were equivalent to Trolox (DPPH IC_50_ 0.018 mg mL^−1^). The DPPH scavenging capacity of different extracts (100% methanol) of *E. arborea* and *C. osmundacea* collected from the Baja California Peninsula has already been reported [16]: the DPPH IC_50_ for these two species were 0.069 and 0.227 mg mL^−1^, respectively, being classified as the most potent among 17 species tested and with a similar or superior scavenging capacity than butylated hydroxytoluene (BHT) (IC_50_ = 0.0867 mg mL^−1^). Differences between these values and our results could be attributed to not only differences in solvents and extraction methodologies but also differences in collection sites and seasons. To our knowledge, DPPH IC_50_ values of *S. compressa*, *P. californica*, and *E. menziesii* extracts are described for the first time in this article.

The DPPH IC_50_ values among the seaweed extracts tested were lower (therefore, better) (0.93–8.07 mg mL^−1^) compared to 80% ethanolic extracts from brown seaweeds (IC_50_ >10 mg mL^−2^) reported by other authors [65]. However, the IC_50_ values observed were higher when compared to the most potent antioxidant seaweed extracts such as: *E. cava* 80% ethanol (IC_50_ 0.01 mg mL^−1^) and *F. vesiculosus* 50–70% ethanol extract (IC_50_ 0.03 mg mL^−1^) [15].

The five macroalgae hydroethanolic extracts were also able to quench oxygen-free radicals in a test tube and showed significant differences among them (Table 3). ORAC scavenging potential of the different species follows a different order than for DPPH; this is due to the presence (in each extract) of different mixtures of antioxidants, with different physicochemical properties or structural features and with different mechanisms of *in vitro* antioxidant activity and therefore different sensitivities for each method [34]. *S. compressa* and *E. arborea* were the most potent species (0.82 and 0.80 mmol TE g dry extract^−1^), followed by *P. californica* (0.54 mmol TE g dry extract^−1^) and *C. osmundacea* and *E. menzziesii* (0.26 and 0.192 mmol TE g dry extract^−1^). The ORAC of the five seaweeds' hydroethanolic extracts, to our knowledge, is described herein for the first time.

ORAC values found in this study were higher than those reported for other brown seaweed species [15]. It must be noted, however, that the ORAC value for *C. osmundacea* was in the range of values reported for other species of the same genus: *C. abies-marina* (0.275–1.314 mmol TE g^−1^) [66].

In this study, no correlation between the five seaweed extracts' TPC and DPPH radical-scavenging activity (*R*^2^ = 0.005) was observed. TPC and extract ORAC values showed a slight correlation (*R*^2^ = 0.36), but this was improved (*R*^2^ = 0.87) when *C. osmundacea* was not considered in the regression. *C. osmundacea* with high phenolic content displayed lower activity than expected, suggesting that other compounds were also responsible for this result. NFE extract content and ORAC radical-scavenging activity also showed a strong correlation (*R*^2^ = 0.91). The two algae extracts with the highest ORAC activities (*S. compressa* and *E. arborea*) were the richest in NFE and were among the richest in polyphenols, suggesting that in both seaweeds, carbohydrates and polyphenols were the main components working as antioxidants. The antioxidant effect of brown seaweed extracts rich in these two major polymeric fractions has been reported by numerous authors [24–26, 34]. The correlation between these compounds and the antioxidant activity is strong for some extracts or inexistent for others. The change in the chemical structure of these compounds during the extraction and the interferences caused by some components present in the extracts are some of the multiple reasons for which there may be variations in the correlation [15, 67, 68].

### 3.4. Glycosidase Inhibition

**Figure 1:**
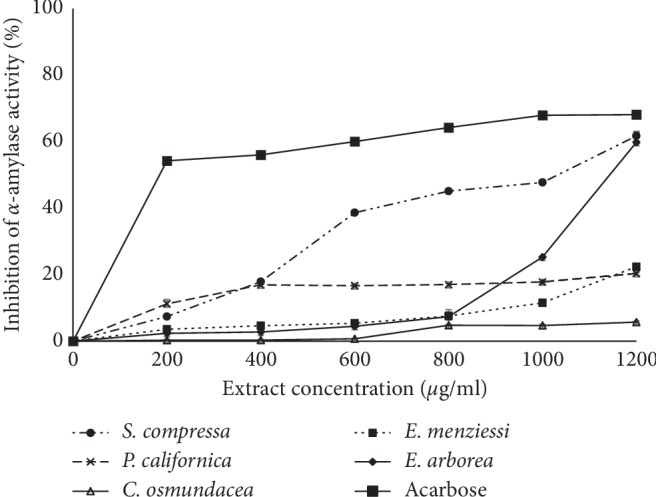
*α*-Amylase inhibitory activities of different concentrations of ethanolic extracts from *Silvetia compressa*, *Cystoseira osmundacea*, *Ecklonia arborea*, *Pterygophora californica*, and *Egregia menziesii*. Data are mean ± SE (*n* = 3).

**Figure 2:**
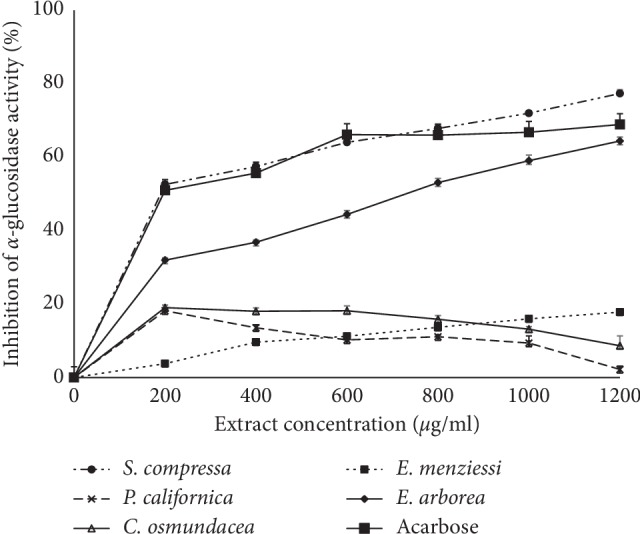
*α*-Glucosidase inhibitory activities of different concentrations of ethanolic extracts from *Silvetia compressa*, *Cystoseira osmundacea*, *Ecklonia arborea*, *Pterygophora californica*, and *Egregia menziesii*. Data are mean ± SE (*n* = 3).

The capacity to inhibit enzymes was different among hydroethanolic seaweed extracts. *S. compressa* and *E. arborea* extracts displayed the highest *α*-amylase and *α*-glucosidase inhibitory activities (the lowest IC_50_) (Figures 1 and 2; Table 3). The rest of the seaweed extracts were inefficient at inhibiting 50% of both glycosidase activities (IC_50_ >1200 *μ*g mL^−1^). The IC_50_ values against *α*-amylase and *α*-glucosidase were 940.1 *μ*g mL^−1^ and 194.2 *μ*g mL^−1^, respectively, for *S. compressa* extract, and 1151.8 and 646.8 *μ*g mL^−1^, respectively, for that of *E. arborea*. Values found for acarbose, the positive control in this study (152.9 and 184.1 *μ*g mL^−1^ respectively), were close to values observed in previous studies [29, 32]. In comparison to acarbose, *S. compressa* and *E. arborea* showed poor inhibitory efficiencies against *α*-amylase (16 and 13%). In contrast, *S. compressa* extract was almost as effective as acarbose (95%) in inhibiting glucosidase, while *E. arborea* extract was less effective, displaying 28% of the acarbose activity. To our knowledge, *α*-glucosidase or *α*-amylase inhibitory activity of the five seaweed extracts evaluated in this work is described here for the first time.

Currently, there is not much information about other *Silvetia* species' capacity to inhibit amylase activity; an IC_50_ value reported for a *Pelvetia caniculata* ethanol : water (80 : 20) extract (51.0 *μ*g/mL) was almost twenty times lower [19]. In contrast, *Ecklonia* species have been recognized as good sources of carbohydrase inhibitors. Moon et al. [69] examined the effect of methanolic extracts (ethyl acetate fraction) from two *Ecklonia* (*Eisenia*) species, *E. stolonifera* and *E. bicyclis*, on *α*-glucosidase activity and found that their inhibitory effect was substantially stronger than that of acarbose. *α*-Amylase IC_50_ was >500 *μ*g mL^−1^ for *E. bicyclis* (85.3 mg PGE g^−1^ dry weight) [18].

When comparing IC_50_ values of our seaweed extracts with those reported for the ethanolic extracts of other species, *S. compressa* and *E. arborea* hydroethanolic extracts were relatively ineffective as *α*-amylase inhibitors. For example, IC_50_ values for *A. nodosum*, *F. serratus*, *F. vesiculosus*, *P. caniculata* and *F. spiralis* ethanolic extracts (80 : 20) were 10 to 20 times lower: 44.7, 70.6, 59.1, 51.0, and 109 *μ*g mL^−1^, respectively [19].

The potency of the *α*-glucosidase inhibition produced by *S. compressa* extract was 2 to 22 times higher than that produced by other brown seaweed species, such as *P. arborescens* (IC_50_ 260 *μ*g mL^−1^, water extract) and *H. macroloba* (IC_50_ 4220 *μ*g mL^−1^, water extract) [17]. Nonetheless, *S. compressa* extract was not as efficient as *F. vesiculosus* ethanolic extract, one of the most potent glucosidase inhibitor seaweed extracts studied to date, whose IC_50_ is <0.5 *μ*g mL^−1^ [19]. As previously mentioned, environmental differences, seasonal variations, differences in the extraction methods, and the degree of purity of the extracts, as well as the chemical structure and molecular weight of the active compounds may explain the differences in the potency of seaweed-derived extracts.

Since we did not obtain *α*-amylase and *α*-glucosidase IC_50_ values for every seaweed extract, the IC_10_ was calculated for correlation analysis with the chemical compounds present in the extracts (Table 3). With these values, a negative linear correlation was found for *α*-amylase with NFE (*R*^2^ = 0.54) and for *α*-glucosidase IC_10._ A negative potential correlation was found with TPC (*R*^2^ = 0.89), as well as a slight polynomial correlation with soluble polysaccharides (*R*^2^ = 0.67). Indeed, the *S. compressa* extract with the best amylase and glucosidase IC_50_ was the one richest in TPC and second richest in carbohydrates. On the other hand, *E. arborea* extract, with the second-best amylase and glucosidase activities, was the seaweed richest in carbohydrates. The ability of naturally occurring polyphenols from brown seaweeds to inhibit enzymes, including *α*-amylase and *α*-glucosidase, has been widely reported [14, 19, 69, 70]. Furthermore, a number of these studies have demonstrated a strong correlation between phenolic content, enzyme inhibition, and antioxidant properties [19]. Recently, it has been shown that ethyl acetate fractions obtained from brown seaweeds, rich in oligomers of phloroglucinol, evidenced a pronounced inhibitory effect on *α*-amylase and *α*-glucosidase activities [18, 29].

Water soluble carbohydrates (e.g., fucoidan) found in great abundance in our active seaweed extracts, mainly in *E. arborea*, have also been reported as potent carbohydrase inhibitors [32, 67]; the synergy of these two types of compounds has also been suggested. Interestingly, the enzyme inhibition activity in our extracts was lost when polysaccharides were removed from the extract by precipitation with ethanol (results not shown). Therefore, the synergistic effect of different compounds present in the hydroethanolic extract deems further research.

### 3.5. Phlorotannin Quantification and Identification

**Figure 3:**
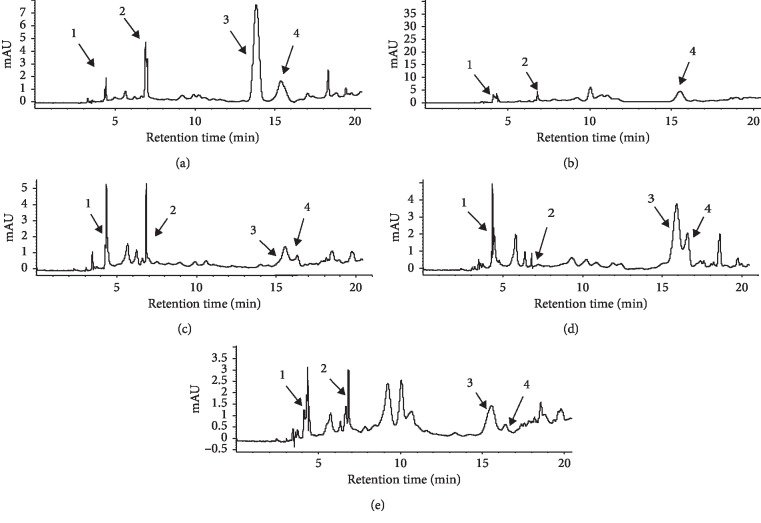
Chromatograms obtained at 270 nm from the ethanolic crude extract of (a) *Silvetia compressa*, (b) *Ecklonia arborea*, (c) *Cystoseira osmundacea*, (d) *Egregia mensienzii*, (e) *Pterigophora californica*. (1) Phloretol; (2) fucophloretol; (3) two units of phloroglucinol; (4) three units of phloroglucinol.

It has been demonstrated that phlorotannins made up more than 82% of the crude polyphenol fraction in a variety of brown seaweeds [71]. Phlorotannins are oligomers or dehydropolymers of phloroglucinols commonly known as marine algal polyphenols [71]. Main phlorotannins were detected in a positive mode (M + H) and included its protonated ions (M + H)^+^, a few of them which were present as sodium or potassium adducts. HPLC-MS-TOF allowed the identification of four main compounds in the *m*/*z* range of 190 to 377 *m*/*z* (Table 4). To our knowledge, this is the first report of phlorotannins in our five seaweeds. The main phlorotannins detected in the seaweed extracts were phloretol, fucophloroethol, and phlorotannins with two- and three-PGU (Figure 3, Table 4). These compounds were found in all seaweed hydroethanolic extracts, with the exception of two-PGU phlorotannin that was not present in *E. arborea*. The concentration of the sum of the four major phlorotannins evaluated was highest for *S. compressa* (723.9 *μ*g PGE per mL of extract), followed by *E. menziesii* (232.1 *μ*g PGE per mL of extract), *E. arborea* (179.7 *μ*g PGE per mL of extract), *C. osmundacea* (101.4 *μ*g PGE per mL of extract), and *P. californica* (72.5 *μ*g PGE per mL of extract). Phlorotannins identified in the present study have been previously reported in other brown seaweeds [14, 20–22].

Among the identified phlorotannins in the extracts, only a negative linear correlation was found between phloretol and DPPH (*R*^2^ = 0.69) and a positive linear correlation was found between three-PGU units of phlorotannin content and ORAC (*R*^2^ = 0.62), reinforcing the fact that NFE was also responsible of antioxidant activity. *α*-Amylase IC_10_ did not show any correlation with phlorotannins. In contrast, *α*-glucosidase IC_10_ showed strong negative linear correlation with the sum of phlorotannins (*R*^2^ = 0.78), with fucophloretol (*R*^2^ = 0.73) and a very high correlation with three-PGU phlorotannin (*R*^2^ = 0.98) contents, but this happened only when *E. menziessi* data were not considered in the regressions. *E. menziesii* extract stands out from other seaweed extracts because of its very low phenolic content but relatively strong antioxidant activity, higher than expected, suggesting that other compounds were also responsible for these actions. A distinct inverse correlation between phenolic contents of *A. nodosum* and IC_70_ for *α*-glucosidase inhibition was also observed by Apostolidis and Lee [70].

Dieckol, 8,8′-bieckol, phlorofucofuroeckol, fucophloroethol, and phlorotannins with low PGU are the main phlorotannins reported as antioxidants and potent *α*-amylase and *α*-glucosidase inhibitors [24, 25, 29]. Fucofuroeckol A and dioxinodehydroeckol have been previously reported as potent carbohydrase inhibitors for *Eisenia bicyclis* [18]. Phloroglucinol was described by Moon et al. [69] as inhibiting 50% *α*-glucosidase at 0.017 mg mL^−1^, and Andrade et al. [72] also related the presence of phloroglucinol (0.23 mg dry algae mL^−1^) in *C. tamariscifolia* with the inhibition of glucosidase. In this study, the samples with the highest content of three-PGU pholorotannin, *S. compressa* and *E*. *arborea*, were the best inhibitors of this enzyme (Table 4).

*S. compressa* extract, the strongest *α*-glucosidase inhibitor in this study, had the highest concentration of phlorotannins, fucophloroetol, and two- and three-PGU phlorotannins.

### 3.6. Sulfated Polysaccharides Identification

**Figure 4:**
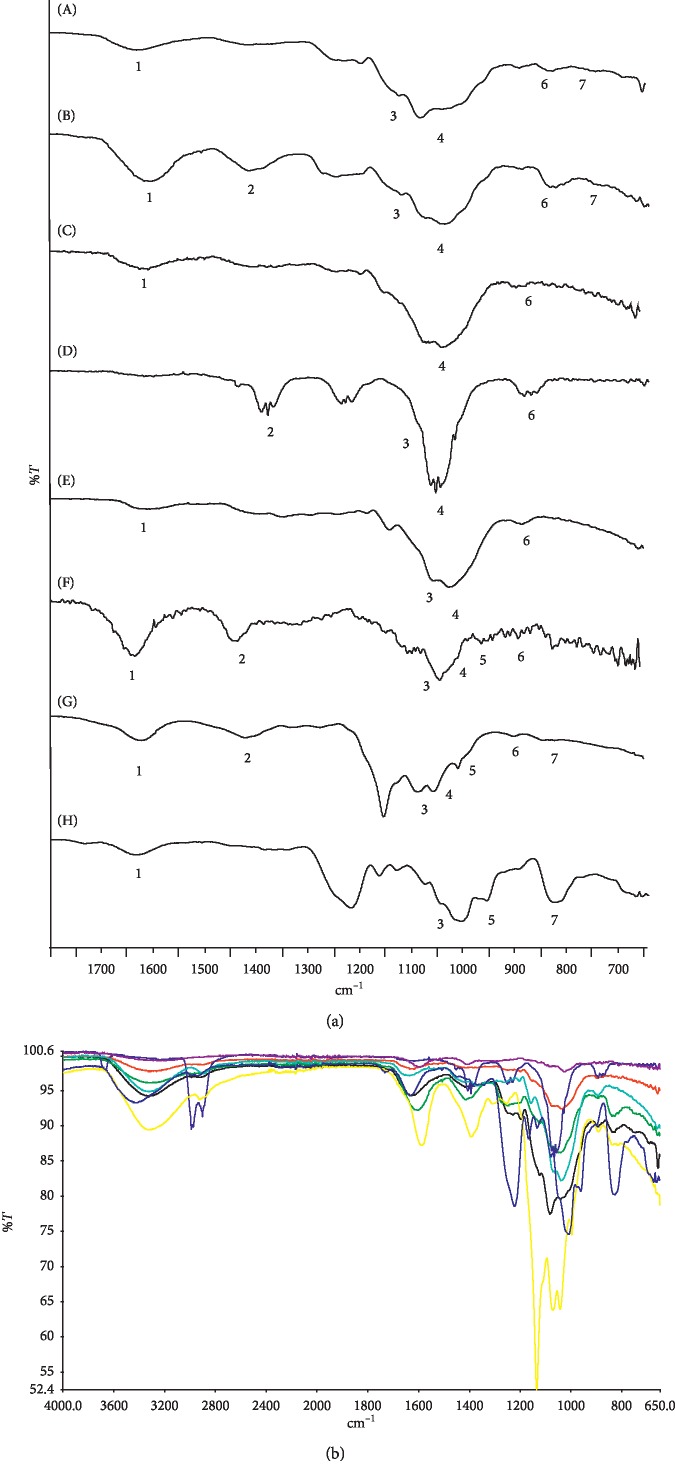
(a) ATR-FT-IR spectra of seaweed extract samples and polysaccharide standards: (A) *Cystoceira osmundacea*, (B) *Silvetia compressa*, (C) *Pterygophora californica*, (D) *Egregia menziessi*, (E) *Ecklonia arborea*, (F) sodium alginate, (G) laminarin, and (H) fucoidan. Numbers 1 to 7 in FTIR spectra indicate most characteristic bands. (b) ATR-FT-IR spectrum of experimental seaweed extracts, sodium alginate, laminarin, and fucoidan standards: *C. osmundacea* seaweed extract (black line); *S. compressa* seaweed extract (green line); *P. californica* seaweed extract (red line); *E. menziesii* seaweed extract (royal blue line); *E. arborea* seaweed extract (aqua line); sodium alginate standard (purple line); laminarin standard (yellow line); fucoidan standard (navy line).

Infrared absorption frequencies corresponding to functional groups of different seaweed extracts are presented in Table 5. Sodium alginate, laminarin, and fucoidan standards were compared with the spectra of different seaweed extracts in the region between 3700 and 650 cm^−1^ (Figures 4(a) and 4(b)). Particularly, in our *E. menziesii* sample, no carbonyl group stretching frequency (1616–1732 cm^−1^) was observed as in the rest of the samples and standards, since this extract had the lowest polysaccharide content. Additionally, *E. menziesii* spectra did not show the characteristic signal around 3400 cm^−1^ corresponding to sulfated polysaccharides. Asymmetrical bending vibration (1404–1417 cm^−1^) of CH_3_ and O-H was not observed in *S. compressa* and *C. osmundacea* extracts. A stretching vibration of sulfoxides (S=O) and CN stretching (1028–1083.6 cm^−1^) and sulfate groups at the axial C4 position and C-O-S symmetrical stretching vibrations (824.84–893.38 cm^−1^) were present among seaweed samples: *E. menziesii*, *S. compressa*, and *E. arborea* showed the strongest peaks. Results observed are in agreement with previous studies [73–79]. *S. compressa* showed stronger signals at the frequencies related to sulfate and carbonyl groups compared to *E. arborea*. FT-IR of seaweed extracts showed the characteristic signals of sulfated polysaccharides with different chemical characteristics; therefore, the polysaccharides in combination with phlorotannins in the extracts seem responsible for the antioxidant and enzyme inhibitory effects [34, 80].

## 4. Conclusion

This study demonstrates the nutraceutical potential of brown seaweed species from Baja California. *S. compressa* and *E. arborea* present the highest activities to protect against oxidation, as well as to inhibit enzymes involved in intestinal carbohydrate digestion and assimilation. Their lower inhibitory effect against *α*-amylase activity and stronger inhibition activity against *α*-glucosidase is ideal for managing hyperglycemia with minimal side effects. The correlations between *in vitro* biological activities and extracts' chemical composition suggest that the bioactivities of *S. compressa* and *E. arborea* extracts could be attributed to their high content of polyphenols, phlorotannins (in particular, three-PGU phlorotannin), and associated polysaccharides (fucoidan, laminarin, and alginate). These seaweeds are potential candidates for food products and nutraceutical and pharmaceutical preparations and additives for diabetes management. Moreover, as *S. compressa* and *E. arborea* are edible species, their consumption should be encouraged.

## 5. Future Perspectives

In the future, studies on the optimization of *S. compressa* and *E. arborea* extraction conditions to maximize yields of the active compounds and on the properties of polysaccharides useful for the antioxidant activity and antidiabetic benefits are required. Furthermore, the evaluation of these seaweeds and their extracts as antidiabetic agents both at preclinical and clinical levels is imperative for material application in functional food and pharmaceutical industries.

## Figures and Tables

**Table 1:** Proximal composition (% dry matter, except for moisture) and total phenolic content (TPC as g PGE per 100 g dry matter) of five brown seaweeds collected from Baja California.

	Moisture^*∗*^	Ash	Lipid	Protein Nx6.25	NFE^*∗∗*^	TPC
*S. compressa*	10.9 ± 0.1b	24.9 ± 0.9b	2.93 ± 0.10c	10.4 ± 0.6bc	61.8 ± 0.9b	8.32 ± 0.39e
*C. osmundacea*	10.6 ± 0.1b	33.9 ± 0.5d	1.08 ± 0.07b	9.1 ± 0.1a	55.9 ± 0.3a	3.98 ± 0.17c
*E. arborea*	9.2 ± 0.3a	23.0 ± 0.5a	0.56 ± 0.12a	11.1 ± 0.4cd	65.4 ± 0.5c	5.00 ± 0.18d
*P. californica*	13.4 ± 0.7c	30.1 ± 0.1c	0.55 ± 0.12a	9.6 ± 0.9ab	59.7 ± 1.1b	1.88 ± 0.04b
*E. menziesii*	12.5 ± 0.5c	33.1 ± 1.0d	0.67 ± 0.05a	11.8 ± 0.5d	54.4 ± 1.1a	0.53 ± 0.02a
*F* value	48.890	161.33	317.637	15.867	83.206	109.013
Sig. ANOVA	<0.001	<0.001	0.001	<0.001	<0.001	<0.001

^*∗*^Moisture in the ground seaweed sample; ^*∗∗*^nitrogen-free extract, by difference NFE (100 − ash + lipid + protein contents); different letters in a column indicate different homogeneous subsets as defined by a multiple means comparison test (Tukey).

**Table 2:** Extraction yield (% seaweed dry matter), proximal composition of the extracts (% extract dry matter, except for moisture), and their total phenolic content (TPC as g PGE per 100 g extract dry matter).

Seaweed	Extraction yield	Moisture	Ash	Lipid	Protein Nx6.25	Polysaccharides	NFE^*∗*^	TPC
*S. compressa*	17.5 ± 0.8a	14.7 ± 1.1c	26.4 ± 2.4b	7.0 ± 0.8a	9.3 ± 0.2c	19.7 ± 1.9b	57.3	47.7 ± 2.2e
*C. osmundacea*	19.2 ± 1.7a	10.5 ± 0.3a	59.1 ± 3.7c	12.3 ± 1.1c	4.2 ± 0.1a	11.2 ± 1.0a	24.4	20.7 ± 0.9d
*E. arborea*	32.8 ± 1.9b	19.7 ± 0.4d	17.1 ± 2.7a	5.0 ± 0.5a	7.7 ± 0.5b	53.2 ± 3.9d	70.2	15.2 ± 0.5c
*P. californica*	19.8 ± 1.8a	12.5 ± 0.5b	31.7 ± 3.8b	10.1 ± 0.8b	6.8 ± 0.7b	28.6 ± 2.0c	51.4	9.5 ± 0.2b
*E. menziesii*	20.3 ± 0.3a	12.5 ± 0.7b	52.8 ± 3.2c	9.6 ± 0.6b	9.2 ± 0.5c	5.4 ± 0.9a	28.4	2.6 ± 0.1a
*F* value	106.351	86.139	94.327	40.099	66.684	212.364	—	744.081
Sig. ANOVA	<0.001	<0.001	<0.001	<0.001	<0.001	<0.001	—	<0.001

^*∗*^NFE (nitrogen free extract) calculated by difference.

**Table 3:** Antioxidant activities and half maximal inhibitory concentrations of hydroethanolic seaweed extracts against *α*-amylase and *α*-glucosidase.

Hydroethanolic extract	DPPH IC_50_ mg mL^−1^	ORAC mmol TE g^−1^	*α*-Amylase IC_50_ *μ*g mL^−1^	*α*-Amylase IC_10_ *μ*g mL^−1^	*α*-Glucosidase IC_50_ *μ*g mL^−1^	*α*-Glucosidase IC_10_ *μ*g mL^−1^
*S. compressa*	3.73 ± 0.11c	0.817 ± 0.07c	940.1 ± 8.3a	240	194.2 ± 16.1a	40
*C. osmundacea*	4.20 ± 0.36c	0.257 ± 0.01a	>1200	2000	>1200	105
*E. arborea*	1.67 ± 0.15b	0.801 ± 0.03c	1152 ± 19.9b	840	646.8 ± 0.7b	65
*P. californica*	8.07 ± 0.50d	0.542 ± 0.05b	>1200	170	>1200	105
*E. menziesii*	0.93 ± 0.12a	0.192 ± 0.01a	>1200	920	>1200	420
Acarbose	—	—	152.9		184.1	
*F* value	269.2	218.3	596.7		3148.3	
Sig. ANOVA	<0.001	<0.001	<0.001		<0.001	

PGE: phloroglucinol equivalents; DS: dry seaweed; TE: Trolox equivalents; IC_50_: the half maximal inhibitory concentration. Each value represents the average of three analytical replicates with standard deviation. Different letters (down column) represent significant differences at *P* < 0.05.

**Table 4:** Fragments adducts, *λ* max, and molar mass of most abundant phlorotannins identified by HPLC-DAD and HPLC-MS-TOF methodologies for the hydroethanolic extracts of seaweeds collected from Baja California.

	Peak#	1	2	3	4
	Bioactive compound	Phloretol	Fucophlorethol	Two units of phloroglucinol	Three units of phloroglucinol
	*λ* max (nm)	220, 262	220, 270	220, 268	220, 267
	Molar mass	274.1231	374.0555	252.0809	378.1241
	Fragment adducts (*m*/*z*)	275.1299 M + H, 276.134 M + H + 1 297.1118 M + Na 313.0888 M + K	375.0627 M + H, 376.0679 M + H + 1 377.0613 M + H + 2	253.0876 M + H 254.0904 M + H + 1 275.0719 M + Na	190.0693 M + 2H 190.5667 M + 2H + 1

*μ*g phloroglucinol equivalents ml^−1^ extract	*Silvetia compressa*	19.1 ± 1.5a	71.2 ± 2.5d	480.9 ± 4.7c	152.7 ± 3.2d
	*Cystoseira osmundacea*	37.1 ± 2.1b	24.8 ± 0.8b	29.9 ± 2.1a	14.6 ± 1.4a
	*Ecklonia arborea*	37.1 ± 0.5b	25.1 ± 1.0b	NF	117.5 ± 4.7c
	*Pterygophora californica*	13.2 ± 2.8a	14.5 ± 1.1a	36.0 ± 1.8a	38.8 ± 3.1b
	*Egregia mensienzii*	42.3 ± 2.6b	30.7 ± 1.1c	113.6 ± 2.4b	45.5 ± 1.2b
	References	2	1	3	3
	*F* values	52.5	346.9	5034.7	478.9
	Sig. ANOVA	<0.001	<0.001	<0.001	<0.001

^1^Isaza Martínez and Torres Castañeda [21]; ^2^Steevensz et al. [20]; ^3^Tierney et al. [22]. NF: not found.

**Table 5:** Signals assigned in the ATR/FT-IR second-derivative spectrum of the hydroethanolic extracts from different brown seaweeds and fucoidan, laminarin, and alginate standards.

	Functional groups					
Seaweed/absorption frequency (cm^−1^)	O-H stretching vibrations N-H stretching vibrations (3371–3700 cm^−1^)^1,4,6^	C-H stretching vibrations N-H stretching vibrations (2941–2944 cm^−1^)^1,2,4,7^	Carbonyl group stretching (1616–1732 cm^−1^)^1,5^	Asymmetrical bending vibration of CH3 and OH bending (1369–1420 cm^−1^)^1,3,4,7^	Stretching vibrations of sulfoxides (S=O)-CN stretching (1034–1075 cm^−1^)^1,2,4^	Sulfate groups at the axial C4 position of C-O-S symmetrical stretching vibrations (822–849 cm^−1^)^1,5^

*Silvetia compressa*	3317.11		1603.2	1416.68	1040.32	893.22/828.88
*Cystoseira osmundacea*	3324.58	2924.93	1627.84	1416.73	1083.6/1041.00	893.38/824.84
*Ecklona arborea*	3326.23	2892.87	1635.92		1070/1039.85	893.34
*Pterygophora californica*	3317.17/3695.33		1617.10		1039.84	892.57
*Egregia menziessi*	3239.7/3662.76	2988.65/2972.72/2901.72		1406.59/1394.17	1075.94/1066.32; 1056.99/1028.03	892.97/879.27/869.42
Fucoidan	*3413.56*	*2989.01/2935.16/*	*1634.58*		**1079.42**	*827.51*
Laminarin	*3318.46/3710.74*	*2924.67*			**1071.75**	*892.62/838.90*
Sodium alginate				*1403.89*	**1085**	*877.79*

^1^Lim et al. [73]; ^2^Park et al. [74]; ^3^Yee et al. [75]; ^4^Kannan [76]; ^5^Shekhar et al. [77]; ^6^Guo and Zhang [78]; ^7^D'Souza et al. [79].

## Data Availability

The data (analytical results spreadsheets) used to support the findings of this study are available from the corresponding author upon request.
